# Automatic modeling of student characteristics with interaction and physiological data using machine learning: A review

**DOI:** 10.3389/frai.2022.1015660

**Published:** 2022-11-03

**Authors:** Fidelia A. Orji, Julita Vassileva

**Affiliations:** Multi-User Adaptive Distributed Mobile and Ubiquitous Computing (MADMUC) Laboratory, Computer Science Department, University of Saskatchewan, Saskatoon, SK, Canada

**Keywords:** machine learning, student characteristics, learning interaction data, student physiological data, student modeling, learner characteristics, adaptation

## Abstract

Student characteristics affect their willingness and ability to acquire new knowledge. Assessing and identifying the effects of student characteristics is important for online educational systems. Machine learning (ML) is becoming significant in utilizing learning data for student modeling, decision support systems, adaptive systems, and evaluation systems. The growing need for dynamic assessment of student characteristics in online educational systems has led to application of machine learning methods in modeling the characteristics. Being able to automatically model student characteristics during learning processes is essential for dynamic and continuous adaptation of teaching and learning to each student's needs. This paper provides a review of 8 years (from 2015 to 2022) of literature on the application of machine learning methods for automatic modeling of various student characteristics. The review found six student characteristics that can be modeled automatically and highlighted the data types, collection methods, and machine learning techniques used to model them. Researchers, educators, and online educational systems designers will benefit from this study as it could be used as a guide for decision-making when creating student models for adaptive educational systems. Such systems can detect students' needs during the learning process and adapt the learning interventions based on the detected needs. Moreover, the study revealed the progress made in the application of machine learning for automatic modeling of student characteristics and suggested new future research directions for the field. Therefore, machine learning researchers could benefit from this study as they can further advance this area by investigating new, unexplored techniques and find new ways to improve the accuracy of the created student models.

## Introduction

Artificial intelligence (AI) is a field of computer science concerned with developing smart software systems that mimic human capabilities in understanding and responding to tasks. AI is referred to as “*computing systems that are able to engage in human-like processes such as learning, adapting, synthesizing, self-correction and use of data for complex processing tasks*” (Popenici and Kerr, 2017). The implementation of the capabilities is made possible using AI techniques. The techniques allow inferencing, learning, natural language processing, and perception abilities to be implemented in software systems. Artificial intelligence comprises various strategies and methods, including machine learning in generating, as well as applications of these techniques in solving specific problems. Machine learning is a field of AI which refers to computational methods that improve the performance of a task or make predictions based on available data about the task. The machine learning approach allows software systems to learn from data and to make informed decisions about a specific task based on information learned (Popenici and Kerr, 2017). The approach uncovers valuable insights from data through a combination of concepts from computer science, probability, statistic, and optimization theory. The insights assist in supporting the execution and automation of processes in online educational systems. Typically, data obtained *via* interaction of students with their online educational systems are crucial in predicting their needs, learning patterns, and progress.

Several factors that affect students' academic performance, for example, include emotion, motivation, engagement, and cognitive ability (Yukselturk and Bulut, 2007; Castillo-Merino and Serradell-López, 2014; Cho and Heron, 2015; Lei et al., 2018). Studies reported that emotion, motivation, and engagement have more effect on students' learning with technological systems compared to face-to-face educational settings (Tobarra et al., 2014; Stark, 2019). In the studies, teachers' presence was identified as a mediating factor. This created a need for different methods for automatic assessments of student characteristics as students engage with online educational systems using objective measures (such as students' learning behavior) to be investigated. Being able to predict student characteristics at run-time is crucial for an educational system to automatically and continuously change to adapt to each student's current state. As a result, computer and education researchers are harnessing artificial intelligence (AI) techniques in online educational systems design to make them more engaging, influential, and beneficial.

The increasing learning activities performed on online educational systems results in the generation of a huge volume of learning data. The application of machine learning techniques aimed at harnessing learning data to understand the real learning behavior of students and determine factors that improve learning success is a growing research field. Useful information about learning processes and students' behaviors extracted from learning data is used as input to machine learning techniques for modeling various student characteristics. The role of the characteristics such as student motivation, affective states, and engagement in learning has been recognized by various studies seeking to understand their effect on student learning and performance in different educational contexts. Assessing and identifying the effects of the characteristics on students is important for online educational systems because they have been shown to affect the willingness and ability of students to acquire new knowledge. Prior research has shown that motivated students actively use their learning materials and carry out their assignments (Chan and Ahern, 1999; Schunk et al., 2008). Such students are passionate about their learning activities.

The process of modeling with machine learning techniques produces models which can automatically predict student characteristics based on their learning behavior. Over the past years, several machine learning models targeted at one or more learning characteristics have been developed. The models can broadly be classified into two: models for modeling student characteristics based on physiological data and those based on interaction data. The physiological approach is targeted at behaviors undertaken by students through facial expression, eye-gaze, and neural specificity (Taylor et al., 2004), posture and body language (Boulay, 2011), and wearable sensory devices such as electroencephalography (EEG) and smartwatches (Bauer et al., 2019) are usually employed to capture some of the behaviors. The approach determines behaviors based on complex interactions among physiological, biological, and cognitive systems (Beauchaine, 2001). Examples of student characteristics modeled through the approach include motivation (Taylor et al., 2004), boredom and curiosity (Jaques et al., 2014), and engagement (Monkaresi et al., 2017). Interaction-based approach uses learning interaction data of students to build models for predicting various student characteristics such as valence and arousal (Salmeron-Majadas et al., 2018); motivation (Aluja-Banet et al., 2019); confusion, frustration, and eureka/delight (Graesser et al., 2008); and engagement (Gledson et al., 2021). Each of the two approaches has attracted attention in research.

Interest in developing and using machine learning technology to promote education and learning is increasing. Therefore, it is essential to carry out a literature review on applications of machine learning techniques for modeling various student characteristics automatically. The review will help to identify significant trends, best practices, gaps, and potential opportunities for improving online educational systems using automatic modeling of student characteristics. Additionally, analysis of current literature can help to bring different machine learning techniques, concepts, and processes used for automatic modeling of student characteristics with interaction-based or physiological data together. The modeling process plays an important role in determining the need for learning intervention that can help students.

This review presents novel work by collating current works on automatic modeling of student characteristics through interaction-based and physiological data using machine learning techniques. It answers the following research questions.

RQ1: What are student characteristics targeted for automatic modeling with machine learning techniques?
RQ2: What are effective machine learning techniques for modeling student characteristics based on interaction-based or physiological data?
RQ3: What are the commonly used techniques for capturing students' physiological data?

## Background

### Student characteristics

In order to enhance teaching and learning, a variety of studies discovered and investigated different student characteristics to identify their influence on learning progress and academic performance (Arroyo et al., 2014; Halawa et al., 2016; Liu and Ardakani, 2022). Some of the characteristics include student learning motivation, engagement, affective states, etc. Detecting the characteristics can contribute to supporting and presenting personalized learning content and activities to students. Typically, three different methodological approaches are widely exploited in learning technology research to measure the characteristics: based on self-report, interaction-based, and based on physiological measures. The self-report approach has been widely applied due to its reliability, ease of administration, and validity. The self-report tools vary in length and design and range of construct covered. For example, a tool such as the Motivated Strategies for Learning Questionnaire (MSLQ; Pintrich et al., 1993) integrated expectancy belief, value judgement, affect, and intrinsic/extrinsic orientation so that multiple constructs on student characteristics could be measured. Also, the National Survey on Student Engagement (NSSE; Kuh, 2009) tool is employed in assessing student engagement. Results of self-report tools demonstrated strong links between student characteristics, learning behaviors and academic performance. However, using self-report in assessing student characteristics during learning could be distracting. As a result, current research efforts are geared toward additional methods such as interaction-based and physiological methods that could facilitate dynamic modeling of the characteristics in various learning contexts. Physiological measures assess student characteristics through physical responses such as eye gaze, facial expression, body movement, etc. that occur naturally during learning. Interaction-based measures examine learning logs of students' activities to extract information on learning behaviors patterns, for example, number of accesses to a learning system, time spent, views of contents and assessments, number of assessments completed, etc. are employed in understanding the influence of student characteristics during learning based on context.

Nowadays physiological-based devices capture cognitive and non-cognitive student data to support the building of student models. For example, eye-tracker, cameras for capturing facial expressions, and electroencephalography (EEG) headsets have been used to obtain data employed in building student models that determine their current state during learning processes. Eye-tracking was employed to estimate the concentration level or attention patterns of students during learning process (Conati and Merten, 2007; Joe Louis Paul et al., 2019). The nature of students' concentration was analyzed to provide the basis for supporting and adapting learning components to students. Also, students' facial expressions were captured and analyzed to determine changes in their emotions during learning process (Llanda, 2019; Tonguç and Ozaydin Ozkara, 2020). The studies acknowledge the role of emotion in making students gain more knowledge and in improving their engagement in learning. Furthermore, El Kerdawy et al. (2020) in a study for detecting a student's cognitive profiles used facial expressions and EEG (as physiological monitoring tools) data to build models that detected two cognitive states (instantaneous attention and engagement) and three cognitive skills (planning, shifting, and focused attention). The researchers indicated that EGG and facial expressions provided important features that could be applied for dynamic cognitive state modeling. Consequently, there is a need to review recent works that use the physiological-based approach in a systematic way to provide an overview of the achievements and gaps which can serve as a useful starting point for advancing research in this area.

Furthermore, using interaction-based data for student characteristics modeling is becoming increasingly popular. The main advantage of interaction logs is that sensors are not required in collecting data and data collection does not distract or obstruct students learning processes. The data are captured on online educational systems as students are learning without distraction. Several researchers harnessed learning logs data of students in building models that detected varied student characteristics (Botelho et al., 2017; Hmedna et al., 2020). Information on patterns of student learning behaviors and other learning descriptors were used by the researchers in predicting student states. Thus, the use of log data in generating features for detecting student characteristics has been recognized as a viable approach which can provide a more scalable solution that could be applied in designing and adapting instructional materials to support the needs of students. Therefore, there is a need for a systematic review of current works on the interaction-based approach to analyze and classify scattered research studies in this area under useful headings that could help to identify progress made and aid in advancing research in this area.

A variety of data about students' behavior are being tracked in technology-enhanced learning systems during student learning. Student modeling process employs the tracked data to infer valuable information about student learning patterns, progress as well as characteristics. Student modeling is essential as it identifies the individual needs of the students and their current working context so that learning content and teaching could be adapted based on their needs. Two broad categories of student modeling are static and dynamic modeling. Static student modeling describes a method in which variables used in building student models are initialized just once while the dynamic approach often updates variables used in building student models, enabling a system to react instantly to changes in the examined student characteristics. The dynamic approach enables systems to incrementally learn student characteristics, discover and consider exceptional behavior of students, and respond to changes in a characteristic by updating the student model. The ability of the dynamic modeling process to present a more precise approximation of students' needs is vital for the provision of relevant support to students. Thus, the use of machine learning with physiological and learning interaction data aid in building dynamic student models because student data can be captured at various intervals during learning process. Considering dynamic modeling of student characteristics in technology-enhanced learning can have many benefits for students such as delivering to them adapted learning materials and guidance based on their learning needs.

### Machine learning

Recently, research on developing student models that detect various student characteristics automatically from their learning behavior uses machine learning techniques to facilitate monitoring student behavior during learning process and updating built student models to reflect current states of students. Machine learning is concerned with development of models that enable a system to learn from observational data and draw conclusions automatically. Data acquired by observing students' behaviors and actions during teaching and learning are applied to construct student models. Considerable works have been done in using machine learning techniques to automatically construct student models for various student characteristics. The range of student characteristics investigated in literature includes emotion, motivation, engagement, learning style, and affective states (Botelho et al., 2017; Aissaoui et al., 2019; Hung et al., 2019; Raj and Renumol, 2022; Wang et al., 2022). The most common approach adopted by the majority of these studies is the use of supervised machine learning techniques in creating a model of student characteristics under investigation using features collected from either physiological or interaction log data of students. The studies extracted various features and investigated different machine learning algorithms such as random forest, support vector machine, convolutional neural network, decision tree, etc. Useful results were obtained in the studies with various algorithms and features for several student characteristics. For instance, Hung et al. (2019) automatically discover student emotions using convolutional neural network and facial expressions features. The convolutional neural network model predicted student emotion with an accuracy of 92%.

This review focuses on exploring how various student characteristics are predicted in studies using interaction-based and/or physiological data, thus supervised machine learning approach is the most appropriate method. As a result, we considered only studies that applied supervised machine learning approach to model student characteristics based on interaction-based and/or physiological data. Student characteristics have been identified as an important factor that affects their academic performance—studies that applied supervised approach of machine learning to predict academic performance were excluded (as our focus is on student characteristics modeling and there are other systematic reviews on papers predicting academic performance).

## Materials and methods

Systematic literature reviews relating to a specific research domain provide a means to identify, analyze and understand research gaps and progress in the domain. In this review, we followed the PRISMA guidelines for systematic literature review (Moher et al., 2016), which enabled us to compare, contrast and categorize papers according to different themes and concepts related to student characteristics modeled with machine learning methods. The procedures we followed for the review process were: (1) identified the need to summarize existing evidence on the application of machine learning techniques for automatic modeling of student characteristics, (2) formulated the research questions that the literature review will address, (3) developed a review protocol, (4) identified research databases for our search, (5) identified, selected and saved papers using Mendeley software, (6) screened the papers based on inclusion and exclusion criteria, (7) extracted data from selected papers. (8) performed data synthesis and analysis and (9) reported the review results.

### Resources and search strategy

For our literature search in this review, we used the Elsevier Scopus database, IEEE Xplore, Google Scholar, and the ACM Digital Library for our search. Searching various databases ensures good coverage of studies in this area. We employed the following search items: machine learning and learner model OR machine learning and student model,” “machine learning and learner modeling OR machine learning and student modeling,” and “machine learning and student characteristics OR machine learning and learner characteristics.” Only papers that were published in English between 2015 and 2022 were included in the literature search as we wanted to identify current development and progress made concerning automatic modeling of student characteristics with interaction-based and physiological data using machine learning methods. Moreover, the referenced lists of the selected literature were scanned to find more potentially relevant studies.

### Eligibility criteria for selected papers

The following inclusion and exclusion criteria were defined to ensure that selected papers are in line with the purpose of this review.

### Inclusion criteria

- Peer-reviewed and published research papers.
- Any paper that models one or more student characteristics with machine learning techniques and learning interaction and/or physiological data.
- The paper must report the data types and machine learning algorithms used.
- The paper employed supervised machine learning techniques.

### Exclusion criteria

- Papers on the prediction of academic performance with machine learning techniques and learning interaction and/or physiological data.
- Papers that modeled student characteristics with machine learning but did not use learning interaction or physiological data of students.
- Papers that created student profiles using unsupervised machine learning techniques.
- Duplicate papers with similar contributions by the same author.

### Paper selection process

**Figure 1:**
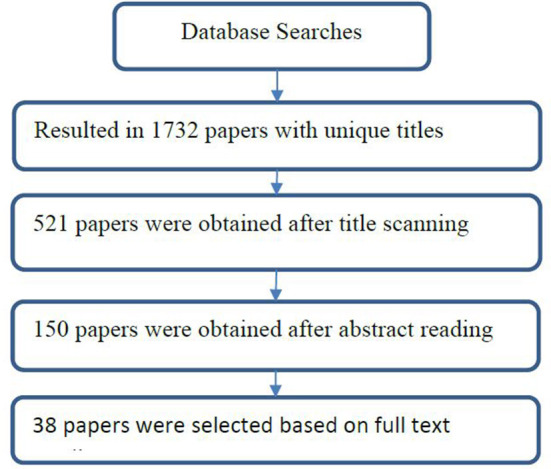
Selection process for the reviewed papers.

Figure 1 summarizes the paper selection process. The search on relevant databases (the Elsevier Scopus database, IEEE Xplore, Google Scholar, and the ACM Digital Library) resulted in 1,732 articles with unique titles. The titles of the articles were screened and those that we considered out of the scope of this study were not selected. Five hundred and twenty-one articles considered relevant were downloaded and their abstracts were read. A total of 150 articles were selected based on review of the abstracts and the exclusion criteria. Furthermore, the full texts of the 150 articles were read and based on our inclusion and exclusion criteria, 38 papers were selected for inclusion in this study. The articles were saved in Mendeley for easy management and referencing.

### Data extraction from the selected papers

For saving extracted data from the articles, we created an excel document with the following columns: Xteristic_modeled, author_name, publication_year, feature_type (interaction or physiological or both), learning_environments (type of systems), number_of_participants, collection_methods (eye-tracker, EEG headsets, cameras, etc.), signal_used (brainwaves, videos of facial expressions, etc.), ML_algorithm_used, and model_accuracy.

### Data synthesis and analysis

Based on the extracted data from the selected articles in an excel document, we analyzed and classified the data into different themes that will help to reveal insights and trends relevant to our research questions.

## Results

The analysis of the selected papers revealed some trends and interesting insights. Our findings are presented below under various categories.

### Data collection and analysis of trend

The results suggest a growing research interest in interaction-based (also called sensor-free) and physiological-based (also referred to as sensor-based) methods for capturing data on students learning behavior over the past few years. Students using online educational systems generate a wealth of data that is stored in log files. The logs record students' behaviors, view counts of different topics and contents, time spent in the system, and other information. Interaction-based features are often derived from the log files containing students' interactions with online educational systems. The interaction-based features used by the studies in this review include view events, assessment attempts, click activities, number of questions answered correctly, number of help requests, navigation order, number of accesses, mouse movements, keystrokes, clickstreams, and time spent on learning. A summary of studies that applied interaction-based features for modeling student characteristics is shown in Table 1. Some of the studies evaluated one machine learning model while a few assessed more than one model. For studies that evaluated a couple of machine learning models, we selected the best-performing models and presented them in Tables 1–3.

**Table 1:** Automatic modeling of student characteristics in learning studies using interaction-based data.

**Studies**	**Number of students**	**Learning environments**	**Features**	**Student characteristic modeled**	**Machine learning models**	**Models accuracy**
Botelho et al. (2017)	646 students	ASSISTments learning platform	Interaction features	Affective states	Recurrent neural network	AUC is 78%
DeFalco et al. (2018)	119 university students	TC3Sim game learning environment	Interaction features	Affective states	Logistic regression	A' is 85%
Ghaleb et al. (2019)	23 university students	Technology-enhanced learning system	Interaction features	Affective states	Support vector machine	Precision is 67%
Hutt et al. (2019)	69,174 high school students	Online math learning platform	Interaction features	Affective states	Genetic algorithm	None
Khan et al. (2019)	81 students	Learning management system (Moodle)	Interaction features	Affective states	Bayesian network	Precision is 67.9%
Salmeron-Majadas et al. (2018)	41 students	Essay writing tool named MOKEETO	Mouse and keyboard interactions	Affective states	Random forest	70%
Wang et al. (2019)	269 undergraduates	Cloud Classroom	Interaction features	Affective states	K*	*F* score is 70%
Edmond Meku Fotso et al. (2020)	3,617 university students	MOOC	Interaction features	Engagement	Recurrent neural network	89.2%
Erkan et al. (2020)	12,447 university students	MOOC (Peer-review participation)	Interaction features, Student performance	Engagement	Random forest	80%
Raj and Renumol (2022)	7,775 university students	Virtual learning environment course	Interaction features, student performance	Engagement	Random forest	95%
Aissaoui et al. (2019)	1,235 students	MOOC	Interaction features	Learning style	Naïve bayes	89%
Amir et al. (2017)	200 university students	Learning management system	Interaction features	Learning style	Support vector machine	83.6%
Hmedna et al. (2020)	52,735 university students	Edx course	Interaction features	Learning style	Decision Tree	98%
Lwande et al. (2019)	199 students	eLearning course	Interaction features	Learning style	K-nearest neighbors	None
Al-Shabandar et al. (2018)	7,000 university students	MOOC (Edx)	Interaction features	Motivation	Decision tree	75.5%
Babić (2017)	129 university students	Learning management system	Interaction features	Motivation	Neural network	76.9%
Abyaa et al. (2018)	48 university students	Online learning platform (piazza)	Interaction features	Personality	Random forest	83.3%

**Table 2:** Automatic modeling of student characteristics in learning studies using physiological data.

**Studies**	**Number of students**	**Learning environments**	**Collection devices/ methods**	**Signal types**	**Student characteristic modeled**	**Machine learning models**	**Models accuracy**
Bixler and D'Mello (2016)	178 undergraduates	Research texts on research methods presented on computer screens	Tobii TX 300 and Tobii T60 eye tracker	Fixations and saccades	Affective states	Bayesian network	72%
Shi et al. (2019)	82 students	MOOC platform	Logitech C920 webcam	Video clips of facial expression	Affective states	Convolutional neural networks and support vector machine	93.8%
Ashwin and Guddeti (2020)	50 students	Classroom environment	Camera	Video clips	Affective states	Convolutional neural networks	95.6%
Hung et al. (2019)	4 university students	Students learning in a class	Camera	Video records	Emotion	Convolutional neural networks	84.6%
Li and Wang (2018)	10 students	Intelligent education system	Camera	Video clips of facial expressions (blink frequency)	Emotion	Convolutional neural networks	Not reported
Liu and Ardakani (2022)	15 students	Affective learning system	EMOTIV EPOC and EEG headset	Brain waves pattern	Emotion	*K*-nearest neighbors	74.3%
Yang and Qi (2020)	70 students	Not reported	Camera	Pictures of students' facial expressions	Emotion	Convolutional neural networks	97%
Booth et al. (2018)	10 students	Interactive computer tasks	EPOC and EEG headset	Brainwave data	Engagement	Random forest	62.5%
Dubbaka and Gopalan (2020)	5 adults	MOOC	Logitech C920 webcam	Video clips of facial expressions	Engagement	Convolutional neural networks	95%
El Kerdawy et al. (2020)	109 university students	Psychology continuous performance tasks	Camera and EEG headset	Video clips of facial expressions and brainwaves data	Engagement	•Random forest (for EEG data) •Convolutional neural networks (for facial data)	86 and 82%
Liu et al. (2018)	8 students	Intelligent class environment	Overhead camera in a wide classroom	Visual focus of attention (VFOA) and head pose estimation	Engagement	Hybrid multilayered random forest	70%
Mohamad Nezami et al. (2020)	20 high school students	Virtual world learning environment named Omosa	Camera	Video clips of facial expressions	Engagement	Convolutional neural networks	72.4%
Monkaresi et al. (2017)	22 students	Students writing essays using google document	Microsoft Kinect Sensor	Videos of their faces and upper bodies (used for estimation of facial expression and heart rate)	Engagement	Naïve Bayes	AUC 0.76%
Aggarwal et al. (2021)	12 undergraduates	MOOC	EEG headset	Brainwave data	Motivation	Support vector machine	94%
Chattopadhyay et al. (2021)	30 students	Game environment	EEG known as brainmarker	Brainwave data	Motivation	Convolutional neural networks	89%
Santos et al. (2020)	45 high school students	Students performing experiments in physics lab	Camera	Video clips of facial expressions	Motivation	Convolutional neural networks	85%
Wang et al. (2022)	25 university students	OGAMA software	Tobii X120 tracker and EEG headset	Fixation, pupil size, saccades, and Brainwave data	Motivation	Logistic regression	92.8%

**Table 3:** Automatic modeling of student characteristics in learning studies using multimodal data.

**Studies**	**Number of students**	**Learning environment**	**Signal types**	**Collection devices/methods**	**Student characteristic modeled**	**Machine learning models**	**Models accuracy**
Altuwairqi et al. (2021)	110 university students	Students performing tasks in a university computer lab	Video clips of facial expressions, mouse movement and keystrokes	Webcam and mouse/keyboard behavior recorders	Engagement	Naïve Bayes	95.2%
Henderson et al. (2020)	119 undergraduates	Game-based learning environment	Interaction features, posture and upper-body movement data	Microsoft Kinect motion tracking sensor	Affective states	Multi-layer perception	89.5%
Lallé et al. (2021)	79 university students	MetaTutor	Eye gaze data and interaction features	Tobii 60 eye tracker	Emotion	Linear regression	48%
Sharma et al. (2020)	32 undergraduates	Web-based self-assessment environment	Interaction features, fixations and saccades, EEG data, video clips of facial expression, and heart rate data	Tobii X3-120 eye tracker, head-mounted portable EEG cap, Logitech webcam, and Empatical E4 wristband	Motivation	Hidden Markov models, support vector machine	90 and 80%

The physiological method uses sensors to record signals of physiological or neurobiological responses of students during learning. The physiological responses involve visible behaviors such as facial expression, eye gaze, and posture based. Cameras were employed in recording videos focused on students' faces as they were learning. Facial action units (FAU), head position, and facial points features were computed from the videos using specialized software such as OpenFace library and Kinect SDK's face tracking engine. For eye gaze, eye trackers (e.g., Tobii eye tracker) were used in capturing fixations and saccades of students' real-time eye responses during learning. To capture students' posture data during learning, Microsoft Kinect motion-tracking sensors were utilized to capture skeletal vertex coordinates based on upper-body movement and posture. The neurobiological responses measure neurobiological changes such as brainwave patterns and heart rates. Wearable EEG headsets were employed in collecting brainwave data. Empatica E4 wristbands were used to measure heart rate, electrodermal activity (EDA), body temperature, and blood volume pulse. An overview of studies that applied physiological data for modeling various student characteristics is presented in Table 2.

Modeling student characteristics using interaction-based or physiological data has shown promising results when used separately, however, studies are investigating whether a multimodal approach (combination of the two data sources) can produce models of student characteristics with improved accuracy. Table 3 lists studies that present a multimodal approach for modeling different student characteristics. Ninety percent (34) of the reviewed papers proposed either interaction-based (17 papers) or physiological methods (17 papers), each accounting for 45% of the total number of papers. A fully multimodal approach, combining interaction-based and physiological methods is the least explored method, employed by only four papers (10% of all the studies) and they are more recent (from 2020 to 2021). The studies that applied the multimodal approach combined eye-tracking and interaction logs; eye-tracking, EEG, wristband, facial expressions, and interaction logs; facial expression, mouse movement and keystrokes; posture-based and interaction logs to build multimodal models for predicting students' affective states, emotion, motivation, and engagement, respectively. Some of these studies revealed that the multimodal method performed better than the model based on a single approach.

### Techniques used with physiological method

**Figure 2:**
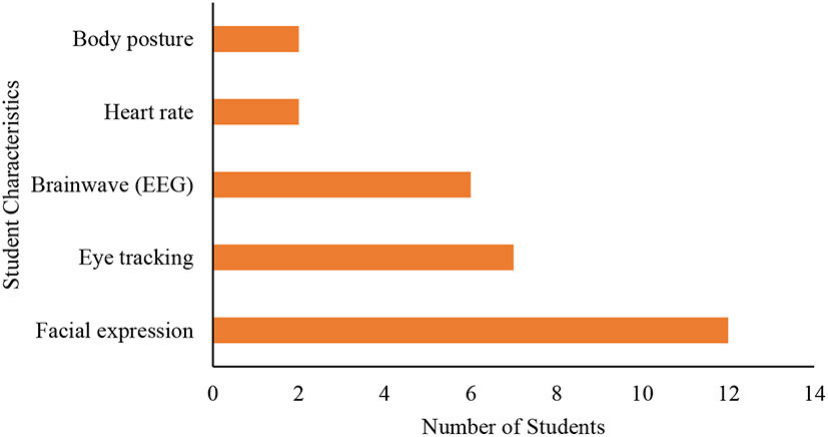
Physiological techniques used in studies.

Figure 2 summarizes the major technological techniques employed by studies that used the physiological approach to model learner characteristics. Some techniques were more often applied than others. For example, among the studies investigated, tracking facial expressions emerged as the most frequently utilized technique (used in 41% of all studies). It is followed by eye tracking (24%) and brainwave measure with EEG (21%). Tracking body posture (7%) and heart rate (7%) are the least employed techniques. The studies that provided their physiological data through them were few and they combined the obtained data with other physiological data sources such as facial expression, eye tracking, and EEG. Generally, tracking facial expressions, EEG, and eye tracking emerged as top techniques that are commonly employed in the physiological method for student characteristics modeling.

### Modeled student characteristics category

**Figure 3:**
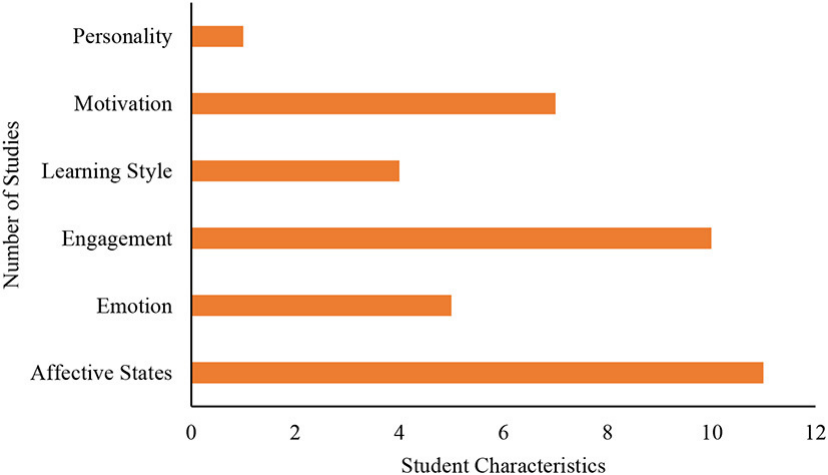
Trend of student characteristics modeled.

As can be seen from Figure 3, the reviewed papers in this study fundamentally focused on modeling automatically six major student characteristics. The characteristics include student affective state, emotion, engagement, learning style, motivation, and personality. Among the 38 studies that modeled different student characteristics using machine learning techniques and interaction-based and/or physiological data, affective states led the list with a total of 29% of all the studies followed by engagement with 26%. Motivation comes third with 18%, and emotion with 13% took fourth place.

### Effective machine learning techniques for modeling student characteristics

Tables 4, 5 summarize various student characteristics modeled using either interaction-based or physiological data and machine learning algorithms. Based on the results from the analyzed studies, effective models capable of predicting students' affective state, emotion, engagement, learning style, and motivation can be developed using interaction-based data. With respect to models' performance, measured as accuracy in predicting different student characteristics, most of the studies analyzed reported impressive model performance (70% and above). Neural networks and random forest models predicted more characteristics effectively with interaction data. Furthermore, the results from the analyzed studies revealed that useful models that can predict students' affective states, emotions, motivation, and engagement can be implemented using physiological data. Interestingly, overall models' accuracy for predicting various student characteristics with physiological data ranges from 62.5 to 97%. Convolutional neural network models effectively predicted more characteristics using physiological data than the other models.

**Table 4:** Student characteristics modeled using interaction-based data.

**Student characteristics modeled**	**Studies that used interaction-based data**	**Machine learning algorithms with effective performance**	**Accuracy of the models**
Affective states		Recurrent neural network, logistic regression, random forest, and K*	Ranges from 70 to 85%
	Botelho et al., 2017; DeFalco et al., 2018; Salmeron-Majadas et al., 2018; Ghaleb et al., 2019; Hutt et al., 2019; Khan et al., 2019; Wang et al., 2019		
Engagement	Edmond Meku Fotso et al., 2020; Erkan et al., 2020; Raj and Renumol, 2022	Recurrent neural network and random forest	Ranges from 80 to 95%
Learning style	Amir et al., 2017; Aissaoui et al., 2019; Lwande et al., 2019; Hmedna et al., 2020	Naïve bayes, support vector machine, and decision tree	Ranges from 83.6 to 98%
Motivation	Babić, 2017; Al-Shabandar et al., 2018	Decision tree and neural network	75.5 and 76.9%
Personality	Abyaa et al., 2018	Random forest	83.3%

**Table 5:** Student characteristics modeled using physiological data.

**Student characteristics modeled**	**Studies that used physiological data**	**Machine learning algorithms with effective performance**	**Accuracy of the models**
Affective states	Bixler and D'Mello, 2016; Shi et al., 2019; Ashwin and Guddeti, 2020	Bayesian networks, convolutional neural networks and support vector machine	Ranges from 72 to 95.6%
Emotion	Li and Wang, 2018; Hung et al., 2019; Yang and Qi, 2020; Liu and Ardakani, 2022	Convolutional neural networks and k-nearest neighbor	Ranges from 74.3 to 97%
Engagement	Monkaresi et al., 2017; Booth et al., 2018; Liu et al., 2018; Dubbaka and Gopalan, 2020; El Kerdawy et al., 2020; Mohamad Nezami et al., 2020	Convolutional neural networks, random forest, and naïve bayes	Ranges from 70 to 95%
Motivation	Santos et al., 2020; Aggarwal et al., 2021; Chattopadhyay et al., 2021; Wang et al., 2022	Support vector machine, convolutional neural networks, and logistic regression	Ranges from 85 to 94%

The variations in the accuracy of the models for each characteristic are probably due to many possible factors (ranging from dataset size, number of features, type of features, and their preparation methods) which could affect machine learning models.

## Discussion

The results of this literature review provide valuable insight for designers of educational systems and researchers about dynamic modeling of various student characteristics. Several machine learning techniques have been employed in detecting student characteristics using learning interaction logs, facial expressions, eye-gaze, electroencephalogram (EEG), and other physiological features.

### Effectiveness of machine learning techniques for modeling student characteristics automatically

Based on the reviewed literature, machine learning techniques are effective at modeling various student characteristics automatically based on interaction-based or physiological data. Among the reviewed papers, 82% reported impressive model accuracy (from 70% and above) in modeling different student characteristics. The results suggest that various student characteristics can be modeled dynamically from their interaction and/or physiological responses during learning.

### Targeted student characteristics for automatic modeling with machine learning techniques

Research indicated that getting useful and reliable student information to support adaptive decision-making is a challenge (Shute and Zapata-Rivera, 2012). This literature review revealed how machine learning methods have been applied to provide useful information about student characteristics which can help in adaptation of teaching and learning. The results of current research trends in Figure 3 revealed six main categories of student characteristics targeted for automatic modeling with machine learning techniques and students' interaction and/or physiological data. The categories include affective states, emotion, engagement, learning style, motivation, and personality. Based on our results, the commonly modeled student characteristics are affective states, engagement, and motivation.

Different researchers define student affective states, engagement, and motivation differently. The term “affective states of students in learning” has been used to denote a range of variables related to their motivation, engagement, and attitude. For example, in Wang et al. (2019), the affective states comprise confusion, engagement, off-task, and frustration. According to Shute and Zapata-Rivera (2012), the affective states consist of motivated, attentive, engaged, and frustrated. Similarly, the term “engagement” has been used to include different affective states by different authors. For instance, Dewan et al. (2019) consider learning gain, delight, boredom, neutral, confusion, and frustration as a scale for engagement levels. Despite the difference in their definition, researchers have the same specific strategies for assessing them. However, motivation is distinct from engagement. Motivation has been characterized as the psychological processes that underpin the energy, purpose, and long-term sustainability of learning activities whereas engagement has been defined as the outward manifestation of motivation (Skinner et al., 2009). Engagement can manifest itself in the form of observable behaviors (for example, involvement in learning, on-task behavior, etc.). “*Therefore, when motivation to pursue a goal or succeed at an academic task is put into action deliberately, the energized result is engagement*” (Wang and Degol, 2014). For instance, Xiong et al. (2015) applied structural equation modeling to MOOC data and revealed that motivation is a strong predictor of student engagement in a course.

Below we discussed the three commonly modeled student characteristics: affective states, engagement, and motivation. The discussion covers studies with high model accuracy and some studies that demonstrated how the models can be used in online educational systems.

### Affective states

Students' affective states during learning impact their interaction, progress, and performance. Detecting positive and negative affective states of students is important in improving and supporting their learning processes and progress. According to research, learning affective states include students' concentration, confusion, frustration and off-task behaviors (Wang et al., 2019). A number of studies conducted in the area of affective computing in education have recognized the role of affective states especially the learning affective states in facilitating learning activities (Wiggins et al., 2015; Jiménez et al., 2018; Standen et al., 2020). For example, Standen et al. (2020) used multimodal sensor data and machine learning to build models that recognized three learning affective states (engagement, frustration, and boredom) and based on the state detected, the presentation of learning contents was adapted to maximize the student's learning rate. The authors used intervention and controlled experiments in evaluating their adaptive system. The results of the evaluation indicated that more engagement and less boredom were experienced in the intervention than in the control session. The results suggest that tailoring learning contents based on students' affective states resulted to increased engagement in learning activities and promoted learning. Liu and Ardakani (2022) collected students' brainwave data using portable electroencephalogram and applied k-nearest neighbor (KNN) machine learning algorithm to recognize affective states in real-time. Based on the recognized states, learning contents were automatically recommended to students. The authors evaluated their system using experimental and control groups. The results of the *t*-test analysis demonstrated that their eLearning system model enhanced students' satisfaction but did not have a significant impact on student engagement and learning. Furthermore, Wiggins et al. (2015) identified students' affective states and learning styles automatically from their learning behavior and preferences within a course. Based on the results of the automatic detection, the researchers developed a tool which teachers can use to identify students' affective states and learning styles. These studies demonstrated that the incorporation of affective states models into online educational systems can be used to improve students learning progress and performance and to assist teachers in understanding their students' conditions in a learning context, thus effective affective states models are important in detecting student's various states on different learning contexts.

### Students' engagement

Student engagement with online educational systems is important in understanding their learning experience with the systems. The ability to recognize engagement levels automatically plays a role in designing and responding to student engagement issues. Student engagement tracking based on interaction and/or physiological data is crucial for the automatic detection of engagement in online educational systems. Data collected *via* these approaches are employed in modeling and designing adaptive learning environments that will support student learning. A variety of studies (Monkaresi et al., 2017; Dubbaka and Gopalan, 2020; Erkan et al., 2020; Raj and Renumol, 2022) reported many students' online learning behaviors and physiological responses that can be extracted and applied in building models that will automatically predict students' engagement levels. The studies reported the accuracy of their models to indicate the extent to which engagement levels could be dynamically detected using their approach. For example, a study by Raj and Renumol (2022) predicted student engagement with an accuracy of 95% using learning interaction data and a random forest model. This study achieved the best performance in terms of accuracy among all the reviewed papers that predicted student engagement levels using learning interaction logs. Hussain et al. (2018) developed a dashboard which incorporated a model of student engagement built using their interaction data, grade scores, and machine learning into an online education system to help instructors in assessing student engagement levels in online courses in relation to various activities and resources, and to offer additional interventions for students prior to their final exam. Dubbaka and Gopalan (2020) reported an accuracy of 95% for the prediction of student engagement using facial expression data and convolutional neural network model. The research achieved the best performance in terms of accuracy among all the reviewed papers that predicted student engagement levels using physiological data. To provide robust and more effective student engagement measures, Altuwairqi et al. (2021) combined learning interaction and physiological data (facial expression data, mouse movement and keystrokes) of students to build a naïve bayes model that predicted student engagement with an accuracy of 95.2%. The accuracy level of the two approaches suggested that automatic student engagement detection can effectively track issues on engagement in learning environments.

Based on this literature review, the most predominant approach for detecting engagement using physiological data is facial expression tracking. A number of studies (Monkaresi et al., 2017; Dubbaka and Gopalan, 2020; Mohamad Nezami et al., 2020; Altuwairqi et al., 2021) analyzed student engagement during interaction with online educational systems using facial expressions. The studies focused on capturing and analyzing facial expressions to identify disengagement and engagement states of students. Facial expression recognition is less invasive and integrated cameras on computer systems are easily accessible in majority of learning platforms.

### Student motivation for learning

Motivation is an important factor that influences students learning. Research has shown that student motivation is a fundamental factor that affects their success (Yukselturk and Bulut, 2007). Muilenburg and Berge (2005) in a survey-based study identified poor motivation as one of the main barriers to students' online learning. Several studies revealed that motivated students are more likely to be actively engaged, embark on challenging tasks, and demonstrate improved performance and outcomes (Chan and Ahern, 1999; Schunk et al., 2008). As a result, the need to detect and support students to overcome motivation issues based on learning context has led researchers to explore various ways student motivation can be predicted automatically during teaching and learning. In line with the need for dynamic modeling of student motivation, studies have demonstrated that student motivation can be predicted from their learning interaction and/or physiological data using machine learning (Al-Shabandar et al., 2018; Santos et al., 2020; Aggarwal et al., 2021). Among the papers on student motivation we reviewed, Babic obtained a better accuracy of 76.9% for predicting student motivation with learning interaction logs and neural network models (Babić, 2017). Wang et al. (2022) reported a better accuracy of 92.8% in predicting student motivation with physiological data (EEG and eye gaze) and logistic regression model. Also, an accuracy of 90% was obtained in predicting student effort using multimodal data (consisting of learning interaction logs, eye-tracking, EEG, wristband, and facial expressions) and Hidden Markov Models (Sharma et al., 2020). These studies have revealed valuable insights into the usefulness of learning interaction logs, physiological data, and machine learning methods for dynamic modeling of student motivation. More details on modeling student motivation and adaptation of teaching and learning based on it are provided in Orji and Vassileva (2021).

In general, considerable progress has been made in using student interaction logs or their physiological data for detecting student affective states, engagement, and motivation in learning through machine learning techniques. The techniques that have been applied in developing various models range from simple (classification) to sophisticated ones (convoluted neural network) that can diagnose and predict student needs during learning process. The transformational potential of modeling techniques facilitates a more accurate estimation of student needs so that appropriate learning intervention could be delivered to support the student. The models have been found useful in designing smarter educational systems that serve better the students, educators, and administrators. The information provided by the models is used by online educational systems to adapt their responses dynamically to each student through providing personalized learning content, help, instructions, and feedback. It is also employed in understanding the effect of different learning processes and behaviors.

The importance of online educational systems in supporting teaching and learning cannot be overemphasized, however, to make the systems more effective and reduce the issues of high attrition rates of students, the need for dynamic adaptation of teaching and learning based on each student's needs has been highlighted. Adaptation is accomplished based on student models and the models identify student needs through their characteristics. Thus, this study identified student characteristics that are being modeled automatically and how. Researchers, educators, and online educational systems designers will benefit from this study as it could be used as a guide for decision-making when creating student models for adaptive educational systems that could detect students' needs during learning processes and adapt appropriate learning interventions based on the detected needs. Moreover, the study revealed progress made in the application of machine learning for automatic modeling of student characteristics. Therefore, machine learning researchers could benefit from this study as they can further advance this area by investigating how to improve the accuracy of the models and other techniques that have not been explored.

### Future work

With advances in computers and human-computer interaction, the use of interaction or physiological data has been shown to be a viable solution for creating adaptive educational systems with suitable algorithms. The current state-of-the-art data acquisition methods and machine learning techniques offer promising opportunities to show that effective student characteristics models can be developed and applied for various decision-making in learning contexts. Thus, there are many opportunities for future research in this area including:

- Further examining various learning features, preparation methods, and machine learning techniques that may improve the accuracy of student characteristics models developed with interaction or physiological data.
- The link between interaction and physiological data features with student characteristics has been established, it will be interesting to investigate if the features generalize across learning contexts.
- Furthermore, few researchers build student models with a combination of different data modalities (interaction and physiological data) to detect a variety of student characteristics. The researchers revealed that multimodal models that combine different feature metrics such as facial expression and interaction data provided improved accuracy than unimodality models. Robust models of student characteristics could be achieved using multimodal data, thus building more effective multimodal models, and determining the extent to which adaptations based on these models enhance learning and performance is an important research direction.

## Conclusion

This paper provides a review of the trends and effectiveness of machine learning for automatic modeling of student characteristics. Our review results demonstrated that interest in automatic modeling of a wide range of student characteristics (especially student affective states, engagement, motivation, learning style, and emotion) using machine learning continues to grow. A research trend on dynamic modeling of the characteristics become prominent in the past years and considerable progress has been made in this area, with the goal of developing more effective models that can identify student needs during teaching and learning to improve adaptivity and student learning. Detecting student characteristics during the learning process and adapting teaching tactics will help to support better student learning. Thus, researchers are investigating automatic modeling methods such as machine learning techniques for dynamically assessing in real-time student characteristics using their digital traces and physiological responses obtained during learning processes with online educational systems. Current advances in technology such as cheap miniature sensors, digital cameras, and machine learning algorithms, enable unobtrusive continuous measurement of physiological and interaction data of students during learning, offering the ability to predict student characteristics during various learning contexts. The ability of the models to detect the precise needs of the students will improve the success of adaptive learning interventions that depend on them. The paper presented an analysis and classification of student characteristics and the most prevailing machine learning algorithms for modeling them over the last 8 years, along with some future trends.

## Data availability statement

The raw data supporting the conclusions of this article will be made available by the authors, without undue reservation.

## Author contributions

Both authors listed have made a substantial, direct, and intellectual contribution to the work and approved it for publication.

## Funding

This work was supported by NSERC through the Vanier Canada Graduate Scholarship CGV-175722 of FO and the Discovery Grant program RGPIN-2021-03521 of JV.

## Conflict of interest

The authors declare that the research was conducted in the absence of any commercial or financial relationships that could be construed as a potential conflict of interest.

## Publisher's note

All claims expressed in this article are solely those of the authors and do not necessarily represent those of their affiliated organizations, or those of the publisher, the editors and the reviewers. Any product that may be evaluated in this article, or claim that may be made by its manufacturer, is not guaranteed or endorsed by the publisher.
